# Comparison of the Erectile Dysfunction Drugs Sildenafil and Tadalafil Using Patient Medication Reviews: Topic Modeling Study

**DOI:** 10.2196/32689

**Published:** 2022-02-28

**Authors:** Maryanne Kim, Youran Noh, Akihiko Yamada, Song Hee Hong

**Affiliations:** 1 College of Pharmacy Seoul National University Seoul Republic of Korea; 2 Research Institute of Pharmaceutical Sciences Seoul National University Seoul Republic of Korea

**Keywords:** topic modeling, patient preference, patient-centered communication, erectile dysfunction, PDE5 inhibitor, phosphodiesterase type 5 inhibitor

## Abstract

**Background:**

Topic modeling of patient medication reviews of erectile dysfunction (ED) drugs can help identify patient preferences regarding ED treatment options. The identification of a set of topics important to the patient from social network service drug reviews would inform the design of patient-centered medication counseling.

**Objective:**

This study aimed to (1) identify the distinctive topics from patient medication reviews unique to tadalafil versus sildenafil; (2) determine if the primary topics are distributed differently for each drug and for each patient characteristic (age and time on ED drug therapy); and (3) test if the primary topics affect satisfaction with ED drug therapy controlling for patient characteristics.

**Methods:**

Data were collected from the patient medication reviews of sildenafil and tadalafil posted on WebMD and Ask a Patient. The latent Dirichlet allocation method of natural language processing was used to identify 5 distinctive topics from the patient medication reviews on each drug. Analysis of variance and a 2-sample *t* test were conducted to compare the topic distribution and assess whether patient satisfaction varies with the primary topics, age, and time on medication for each ED drug. Statistical significance was tested at an alpha of .05.

**Results:**

The patient medication reviews of sildenafil (N=463) had 2 topics on treatment benefit and 1 each on medication safety, marketing claim, and treatment comparison, while the patient medication reviews of tadalafil (N=919) had 2 topics on medication safety and 1 each on the remaining subjects. Sildenafil’s reviewers quite frequently (94/463, 20.4%) mentioned *erection sustainability* as their primary topic, whereas tadalafil’s reviewers were more concerned about *severe medication safety*. Those who mentioned *erection sustainability* as their primary topic were quite satisfied with their treatment as opposed to those who mentioned *severe medication safety* as their primary topic (score 3.85 vs 2.44). The discovered topics reflected the marketing claims of *blue magic* and *amber romance* for sildenafil and tadalafil, respectively. The topic of *blue magic* was preferred among younger patients, while the topic of *amber romance* was preferred among older patients. The topic *alternative choices*, which appeared for both the ED drugs, reflected patient interest in the comparative effectiveness and price outside the drug labeling information.

**Conclusions:**

The patient medication reviews of ED drugs reflect patient preferences regarding drug labeling information, marketing claims, and alternative treatment choices. The patient preferences concerning ED treatment attributes inform the design of patient-centered communication for improved ED drug therapy.

## Introduction

Topic modeling has been used frequently in various health care fields, including clinical research and health communication, for uncovering themes hidden in natural languages. For example, topic modeling has been used to characterize people’s opinions about vaccines communicated on Twitter [1], to predict clinical outcomes using notes on electronic health records [1,2], and to identify patients’ medical conditions from referral letters [3]. Topic modeling has also been applied in pharmacovigilance to identify drugs with similar safety concerns and therapeutic uses based on the Food and Drug Administration (FDA) drug labeling information [4,5].

Recently, topic modeling on data collected via social network services (SNSs), such as Twitter, and web portals is widely used for the survey of public perceptions and attitudes toward the COVID-19 outbreak [6,7], containment strategies [8,9], treatment interventions [6], and vaccines [10,11]. Topic modeling on SNS data is useful for examining issues that change quickly over time [12]. Topic modeling is especially useful for studying private and sensitive issues such as abortion [13], domestic violence [14], and bullies [15]. On SNSs, people freely reveal their honest attitudes and opinions, while being reluctant to do so on formal surveys when their attitudes and opinions contradict social desirability [16,17]. In fact, a recent study reported that adults in mainland China actively search the internet for information on premature ejaculation [18].

The drug reviews on SNSs can be regarded as patient-reported outcomes (PROs) conveyed in natural language. Directly coming from patients without clinician filtering or interpretation, drug reviews represent the treatment effectiveness and medication safety experienced by individual patients [19-21]. Drug reviews therefore likely contain the labeling information approved by the regulatory agency. They also likely include the marketing claims meticulously chosen by sellers to emphasize the treatment benefits. Furthermore, drug reviews may comprise any other information important to the patient whose real-world experience may well be different from that in the trial setting [21,22]. Therefore, the identification of a set of topics important to the patient from SNS drug reviews would inform the design of patient-centered medication counseling, comparative effectiveness research, pharmacovigilance, and marketing.

The 2 erectile dysfunction (ED) drugs sildenafil and tadalafil have been competing as phosphodiesterase type 5 (PDE5) inhibitors for more than 10 years. However, very little is known about what really concerns the patients who take the medication. This study aimed to identify the topics mentioned in SNS drug reviews by patients who had taken an ED drug (sildenafil vs tadalafil). The study’s specific aims were to determine if (1) the topics identified for each ED drug reflect drug labeling information, marketing claims, and other patient concerns; (2) the distribution of primary topics varies with patient characteristics (patient age and time on ED drug therapy); and (3) the satisfaction with ED drug therapy depends on the primary topics controlling for patient characteristics.

## Methods

### Study Design and Settings

Data were collected from the patient reviews on WebMD [23] and Ask a Patient [24] in the United States. Both WebMD and Ask a Patient are health social media that allow patients to browse patient reviews of prescription drugs based on their medication experience and post their own reviews. Patient reviews on WebMD consist of 4 fields. Reviewers can choose a reason for taking the drug, among several possible reasons given by WebMD. There is an open-ended comments section where reviewers can share their treatment experiences, including benefits, medication safety, and how or whether it worked. They can also give their information (optional), such as age and time on medication, by choosing from a list of options. Finally, patients can rate their drug experience in terms of effectiveness, ease of use, and overall satisfaction. The ratings are based on a 5-point Likert scale from 1 (least satisfied) to 5 (most satisfied). Patient reviews on Ask-a-Patient have 8 fields, namely overall satisfaction rating, reason for taking the drug, side effects, comments, gender, age, duration/dosage, and date. Most of the fields are filled manually by the reviewers. They can also rate their treatment based on a 5-point Likert scale provided by the website. To align with the reviews on Ask a Patient, only the overall satisfaction drug rating was selected from WebMD ratings.

### Data Collection

The drug reviews posted prior to July 1, 2019, were collected for both ED drugs. Among the collected reviews on WebMD, posts without any comments were removed. Since Ask a Patient reviews have a separated comments section regarding side effects, the posts without any comments were removed. To exclude spam, we identified and removed the reviews containing “http,” “.com,” or “www.” Reviews by those under the age of 19 years or without age information were also excluded. Reviews by females were not excluded since they may have been written by caregivers or partners who can represent the user’s experience. The reviews were freely available to all web users and did not include private identifiable data. According to the guidelines, in most cases, research involving such reviews is classified as nonhuman research.

### Text Processing

Preprocessing involving tokenization, stop words, stemming, and completion was used to process the content of patient reviews using the R package. A corpus created based on a list of words was then cleaned by removing punctuations, numbers, extra white spaces, and irrelevant words. Typographical errors were also corrected to prevent the errors from being processed as separate words. Bigrams consisting of 2 words frequently appearing together such as “erectile dysfunction” and “side effect” were treated as unigrams before stemming. Stemming was done to reduce inflected words to their word stem. The stemmed words were then replaced with the most prevalently appearing words from the reviews. Finally, a document-term matrix consisting of words along with their frequencies was constructed.

### Topic Modeling

The latent Dirichlet allocation (LDA) method of natural language processing (NLP) was used to discover hidden topics from each set of patient reviews [25]. The algorithm treated each review as a mixture of several topics and each topic as a distribution of words. To identify the correct weights between these matrices, Gibbs sampling was used. For the LDA topic modeling, the number of topics, “5,” was given to each drug. The optimality of the 5 topics was determined based on a density-based method and visualization to find distinctive and independent topics [26-28]. The LDA packages of open-source R were used as the analysis tool. The primary topic was defined as the topic most frequently mentioned in each review [19].

### Drug Labeling Information and Marketing Claims

Drug labeling information for sildenafil and tadalafil was accessed from the drug database of the FDA [29]. The labeling information comprises efficacy, safety, and dosing schedules. Efficacy is measured based on the PROs on erection strength, duration, etc. The evidence on safety documents headaches, nasal congestion, back pain, and muscle pain. Sildenafil has additional safety concerns pertaining to abnormal vision and rash, while tadalafil has an additional safety concern related to pain in the limbs. The approved dosing schedules specify that sildenafil acts for 4 hours as opposed to tadalafil that has an effect up to 36 hours without being affected by food and liquid intake.

With regard to marketing claims, sildenafil was marketed as the “blue pill” or “blue diamond,” with sports stars of the time promoting the slogan “Get back to Mischief.” At the same time, sildenafil was promoted as a recreational aid to expand the consumer base rather than as a medical treatment [30,31]. On the other hand, tadalafil was publicized as fostering a romantic relationship. It was marketed as a drug that makes you ready whenever you feel the urge to make love, especially during weekends, guaranteeing 36 hours of confidence. Furthermore, it was advertised that users can drink and eat while being on the drug [32].

### Statistical Analysis

The frequency of each topic was computed for each review and then summed for all reviews. The Fisher exact test was used to compare the topic distribution between sildenafil and tadalafil. The 2-sample *t* test was performed to test whether the patient medication ratings varied with primary topics, age, and the time on medication between the drugs. Analysis of variance was used to compare the ratings of the medication for each primary topic by age and time on medication. Statistical significance was tested at an alpha of .05.

## Results

### Description of Patient Medication Reviews

The total number of patient reviews posted on Ask a Patient and WebMD was 1567 (547 for sildenafil and 1020 for tadalafil). The number reduced to 1382 (463 for sildenafil and 919 for tadalafil) when ineligible reviews (those without comments, commercial posts, and reviews by those below 19 years of age) were excluded (Table 1). Most of the reviews were from the age group of 45-64 years (ie, 257/463, 55.5% for sildenafil and 559/919, 60.8% for tadalafil). They were mostly written by patients who used the medication for less than a month (163/463, 35.2% for sildenafil and 448/919, 48.7% for tadalafil). Additionally, most reviews were posted by the patients themselves (189/203, 93.1% for sildenafil and 311/343, 90.7% for tadalafil), while a few (less than 4%) were posted by caregivers.

Among the reasons for taking the drug, “Inability to have an erection” was the most common one for both drugs according to WebMD (166/203, 81.8% for sildenafil and 253/343, 73.8% for tadalafil). However, the reason for taking the drug is not clearly distinguished on Ask a Patient since the reviewer has to write manually rather than choose from a list. The reviewers were dominantly males (more than 94% for both drugs); female reviewers were either caregivers or partners of the drug users.

**Table 1:** Characteristics of patient medication reviews.

Demographic			Sildenafil (N=463), n (%)		Tadalafil (N=919), n (%)
**Gender**					
	Male	445 (96.1)		869 (94.6)	
	Female	7 (1.5)		16 (1.7)	
	Not available	11 (2.4)		34 (3.7)	
**Age (years)**					
	19-44	124 (26.8)		225 (24.5)	
	45-64	257 (55.5)		559 (60.8)	
	≥65	82 (17.7)		135 (14.7)	
**Time on medication**					
	<1 month	163 (35.2)		448 (48.7)	
	1 month to <1 year	141 (30.5)		240 (26.1)	
	≥1 year	150 (32.4)		187 (20.3)	
	Not available	9 (1.9)		44 (4.8)	
**Reasons for taking medications (WebMD)^a^**					
	Inability to have an erection	166 (81.8)		253 (73.8)	
	Increased pressure of pulmonary circulation	5 (2.4)		5 (1.5)	
	Pulmonary arterial hypertension	2 (1.0)		1 (0.3)	
	Enlarged prostate	—^b^		22 (6.4)	
	Enlarged prostate with urination problems	—^b^		10 (2.9)	
	Other	30 (14.8)		52 (15.2)	
**Reviewer type (WebMD)^a^**					
	Caregiver	3 (1.5)		13 (3.8)	
	Patient	189 (93.1)		311 (90.7)	
	Not available	11 (5.4)		19 (5.5)	
**Year**					
	2001-2004	21 (4.5)		—^b^	
	2005-2009	201 (43.4)		343 (37.3)	
	2010-2014	179 (38.7)		424 (46.1)	
	2015-2019	62 (13.4)		152 (16.5)	

^a^Only the reviews posted on WebMD have this information.

^b^Not available.

### Identification of Distinctive Topics of Patient Medication Experiences

The number of distinctive topics identified was 5 for each ED drug (Textbox 1). The identified topics were subjectively named based on the top 30 most frequently appearing words. They represented treatment benefits such as *sexual performance* for tadalafil and sildenafil, and *erection sustainability* for sildenafil. They also reflected marketing tags such as *blue magic* for sildenafil and *amber romance* for tadalafil. As for medication safety, sildenafil had a topic named *medication safety* for which events are known to be typical of PDE5 inhibitors, while tadalafil had 2 topics named *mild medication safety* and *serious medication safety*. *Alternative choices*, which is the only topic representing patient concern outside drug labeling information, was identified in both ED drugs.

In addition to the topic of *alternative choices*, *sexual performance* was demonstrated for both ED drugs. As for the topics on medication safety, they were identified in both ED drugs but with different grades, that is, typical safety for sildenafil, and serious and mild safeties for tadalafil. *Erection sustainability* was only observed with sildenafil. As for the topics related to the marketing claims, *blue magic* and *amber romance* were identified accordingly for the respective drugs.

**List of 5 topics and their member words (top 30 frequently appearing words) identified for each drug.**

**Sildenafil (N=461)**

**- Sexual performance (n=102, 22.1%)**

Words: erect, get, hard, wife, can, result, good, orgasm, still, experience, longer, best, without, cut, penile, need, increase, notice, enough, stay, stimulated, keep, taken, rock, ejaculate, flush, morning, since, position, and sexual

**- Erection sustainability (n=94, 20.4%)**

Words: time, last, sex, pill, first, great, long, get, start, doctor, make, got, back, month, little, medical, life, week, morning, love, problem, always, recommend, couple, worth, ever, help, made, stop, and way

**- Medication safety (n=104, 22.6%)**

Words: headache, drug, flush, work, feel, nose, face, eye, stuffiness, slight, mild, red, light, vision, blue, sometime, think, congested, side effect, nasal, pressure, facial, less, nothing, stuff, head, say, drink, seems, and well

**- Alternative choices (n=71, 15.4%)**

Words: Viagra, work, use, trial, side effect, year, problem, Cialis, cause, give, erectile dysfunction, well, intercourse, help, pain, take, blood, due, another, full, generic, perform, sex, year old, high, maintain, wait, find, gave, and never

**- Blue magic (n=90, 19.5%)**

Words: take, hour, effect, like, day, dose, took, much, half, heart, minute, stomach, felt, night, later, know, want, within, start, better, min, bad, beat, med, several, tablet, usual, around, away, and rapid

**Tadalafil (N=915)**

**- Sexual performance (n=166, 18.1%)**

Words: erect, get, hard, sex, wife, can, like, problem, long, need, night, start, enough, able, better, longer, orgasm, sometime, good, keep, life, several, occasion, lot, penile, love, minute, full, perform, and quit

**- Serious medication safety (n=244, 26.7%)**

Words: pain, day, back, leg, bad, lower, severe, ache, sleep, never, worth, muscle, cramp, symptom, stop, still, extreme, away, due, upper, right, thigh, terrible, though, hip, way, ever, like, walk, and neck

**- Mild medication safety (n=181, 19.8%)**

Words: take, side effect, drug, effect, dose, experience, erectile dysfunction, flush, cause, start, eye, great, help, much, mild, bodies, dosage, side, blood, issue, however, increase, read, recommend, taken, heart, wonder, continuation, face, and sore

**- Alternative choices (n=182, 19.9%)**

Words: Cialis, work, use, trial, year, Viagra, week, well, doctor, month, make, good, result, medical, best, Levitra, see, couple, nothing, gave, vision, anyone, later, sexual, since, always, guy, happen, per, and generic

**- Amber romance (n=142, 15.5%)**

Words: time, hour, last, headache, took, pill, first, feel, morning, half, tablet, night, like, great, got, slight, heartburn, nose, thing, notice, felt, stuffiness, give, still, went, much, three, within, think, and weekend

### Primary Topics by Age and Time on Medication

**Figure 1:**
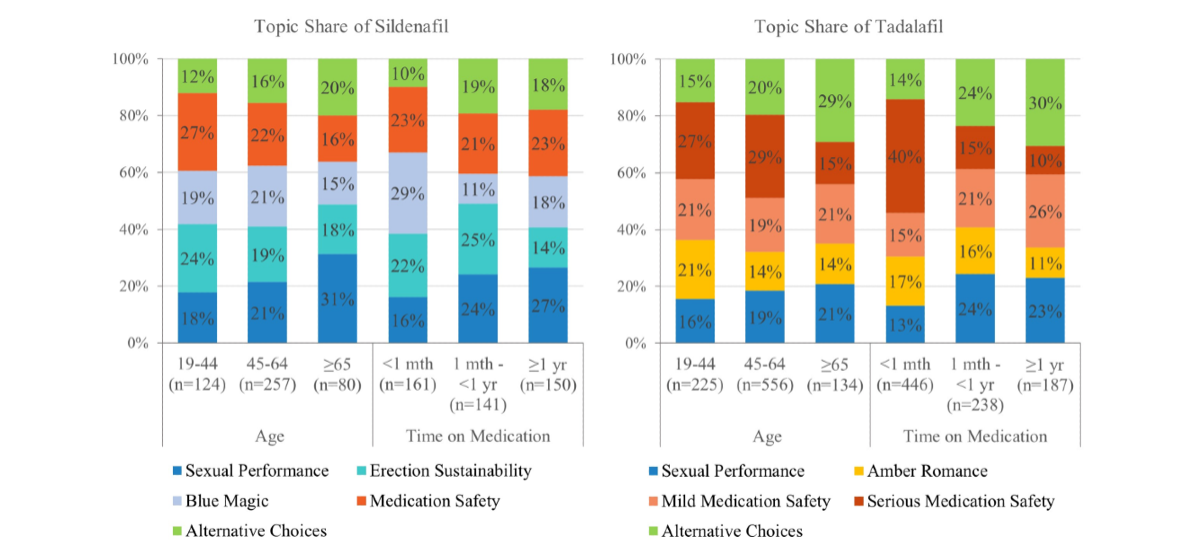
Topic distribution of erectile dysfunction therapy by age and time on medication. mth: month; yr: year.

The topic identified would be primary if it occurs most frequently in a patient medication review. The shares of primary topics varied with patient characteristics (Figure 1). The oldest reviewers of sildenafil most frequently mentioned *sexual performance* as the primary topic, followed by *alternative choices*. However, the oldest tadalafil reviewers most frequently mentioned *alternative choices*, followed by *mild medication safety*. As the reviewers’ age increased, *sexual performance* and *alternative choices* were more likely the primary topics, and *medication safety* was less likely the primary topic. *Medication safety* and *erection sustainability* were most likely the primary topics among youngest sildenafil reviewers, while *serious medication safety* and *amber romance* were most likely the primary topics among youngest tadalafil reviewers.

The most frequently occurring topic did vary with time on the ED drug. Reviewers who experienced the longest time on sildenafil more likely mentioned *sexual performance*. However, those who experienced the shortest time on sildenafil more likely mentioned *medication safety*. The patient reviewers with the shortest time on tadalafil also most likely mentioned *medication safety*, specifically *serious medication safety*. In contrast, those with the longest time on tadalafil least likely mentioned *serious medication safety*.

### Drug Ratings by Primary Topic, Age, and Time on Medication

Drug ratings depended on what topic the reviewers would most likely mention (*P*=.02 for sildenafil and *P*<.001 for tadalafil). Those who mentioned *sexual performance* or *erection sustainability* as their primary topic gave higher ratings than those who mentioned *medication safety* as their primary topic. The dependency of the drug ratings on each primary topic further varied with age as well as time on medication (Tables 2 and 3). Among the sildenafil reviewers, the primary topic of *erection sustainability* had the largest variation in drug ratings across different ages (4.37 for the youngest group compared with 2.86 for the oldest group), followed by the primary topic of *alternative choices* (4.33 for the youngest group compared with 2.94 for the oldest group). The least variation in drug rating across different ages was observed with the primary topic of *blue magic*, which indicates that those reviewers mentioning the primary topic of *blue magic* gave consistent drug ratings regardless of age. Among the sildenafil reviewers, those with the primary topic of *medication safety* had the reverse order of drug rating across ages, with the youngest group giving the lowest rating of 2.56. However, among the tadalafil reviewers, age variation was not apparent, except for the primary topic of *sexual performance*. The youngest group with the primary topic of *sexual performance* gave a rating of 4.17, while the oldest group gave a rating of 3.29. Those with the primary topic of *serious medication safety* reported a drug rating of 2.5 or less across age groups, whereas those with the primary topic of *mild medication safety* reported a drug rating of 3.23-3.64.

**Figure 2:**
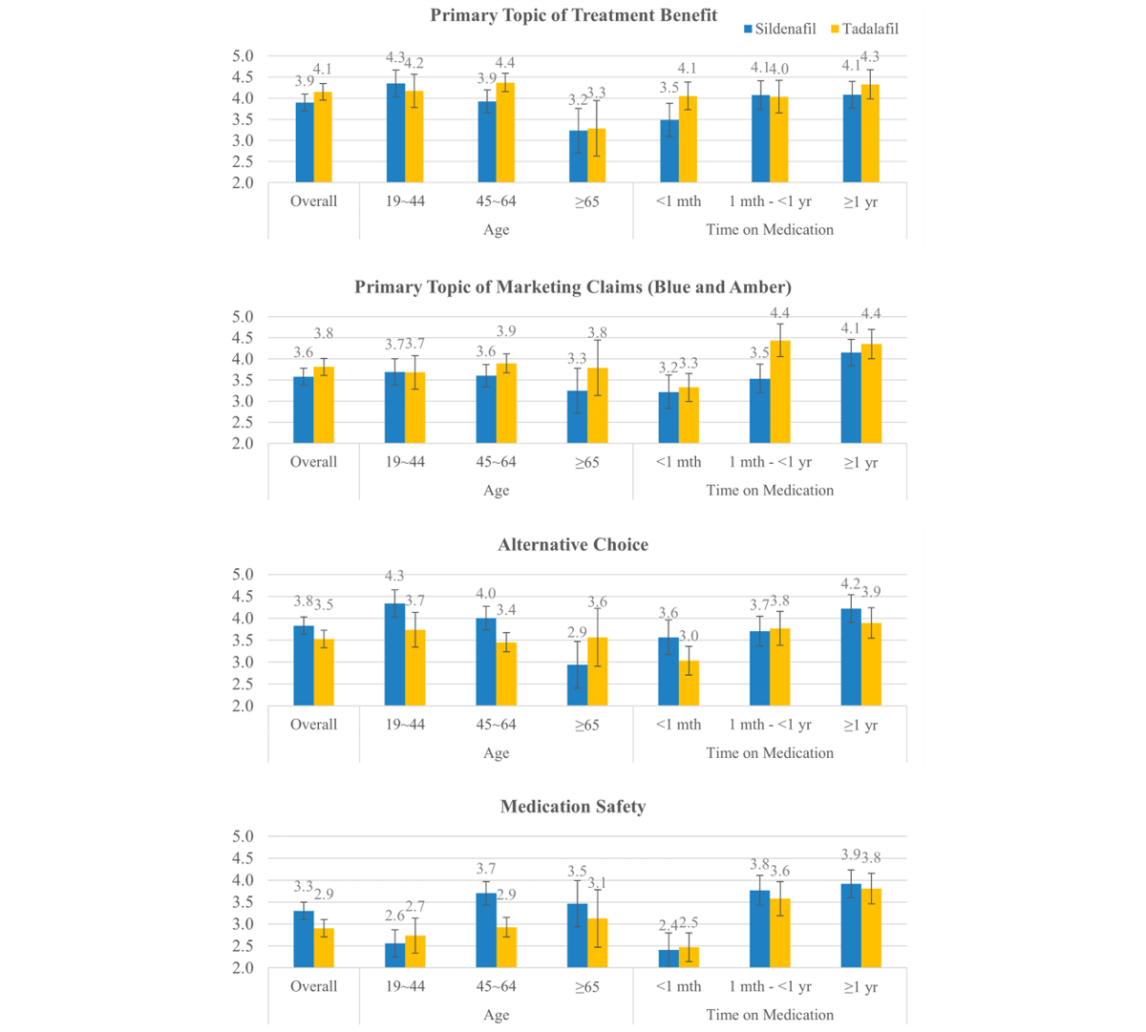
Comparison of treatment satisfaction by primary topics between sildenafil and tadalafil. mth: month; yr: year.

When comparing the drug therapy, medication reviewers rated sildenafil 0.29 points (*P*<.001) higher than tadalafil (Figure 2). Medication reviewers who mentioned topics about treatment benefits, such as *sexual performance* and *erection sustainability*, as their primary topics rated sildenafil lower than tadalafil (3.90 vs 4.14). However, reviewers who mentioned *medication safety* as their primary topic gave the lowest drug rating to each drug, and tadalafil received a lower rating compared with sildenafil (3.30 vs 2.90). Among those who mentioned marketing claims as their primary topic, tadalafil was rated better than sildenafil (3.58 vs 3.81).

When the drug ratings were examined by age and time on medication, the oldest reviewers gave tadalafil a slightly better rating, while younger reviewers gave sildenafil a better rating. Patients aged between 19 and 44 years gave about 0.42 points more for sildenafil than for tadalafil. A longer time on medication was associated with a better rating for the ED drug regardless of drug therapy.

**Table 2:** Drug ratings of sildenafil by age and time on medication.

Variable	Overall rating, mean (SD)	Rating by age (years), mean (SD)				Rating by time on medication, mean (SD)			
		19-44	45-64	≥65	*P* value	<1 month	1 month to <1 year	≥1 year	*P* value
Sexual performance	3.94 (1.42)	4.32 (1.25)	4.02 (1.38)	3.44 (1.56)	.09	3.27 (1.73)	4.26 (1.33)	4.08 (1.16)	.02
Erection sustainability	3.85 (1.42)	4.37 (1.03)	3.82 (1.40)	2.86 (1.75)	.004	3.64 (1.42)	3.89 (1.49)	4.10 (1.37)	.50
Blue magic	3.58 (1.50)	3.70 (1.40)	3.60 (1.53)	3.25 (1.60)	.70	3.22 (1.53)	3.53 (1.55)	4.15 (1.29)	.04
Medication safety	3.30 (1.49)	2.56 (1.58)	3.70 (1.39)	3.46 (0.97)	.001	2.41 (1.42)	3.77 (1.45)	3.91 (1.09)	<.001
Alternative choices	3.83 (1.51)	4.33 (1.40)	4.00 (1.38)	2.94 (1.65)	.02	3.56 (1.75)	3.70 (1.64)	4.22 (1.09)	.28
Total	3.68 (1.48)	3.73 (1.53)	3.82 (1.42)	3.20 (1.52)	.004	3.16 (1.59)	3.88 (1.48)	4.08 (1.18)	<.001

**Table 3:** Drug ratings of tadalafil by age and time on medication.

Variable	Overall rating, mean (SD)	Rating by age (years), mean (SD)				Rating by time on medication, mean (SD)			
		19-44	45-64	≥65	*P* value	<1 month	1 month to <1 year	≥1 year	*P* value
Sexual performance	4.14 (1.29)	4.17 (1.15)	4.37 (1.12)	3.29 (1.70)	<.001	4.05 (1.27)	4.03 (1.47)	4.33 (1.13)	.48
Amber romance	3.81 (1.45)	3.68 (1.38)	3.89 (1.45)	3.79 (1.69)	.73	3.32 (1.53)	4.44 (1.12)	4.35 (0.99)	<.001
Mild medication safety	3.52 (1.52)	3.23 (1.61)	3.64 (1.49)	3.57 (1.43)	.30	3.07 (1.53)	4.06 (1.25)	4.00 (1.27)	<.001
Serious medication safety	2.44 (1.46)	2.34 (1.28)	2.47 (1.51)	2.50 (1.64)	.84	2.24 (1.39)	2.92 (1.42)	3.32 (1.73)	<.001
Alternative choices	3.53 (1.59)	3.74 (1.54)	3.45 (1.62)	3.56 (1.57)	.65	3.03 (1.82)	3.77 (1.41)	3.89 (1.37)	.005
Total	3.39 (1.59)	3.31 (1.54)	3.42 (1.61)	3.39 (1.62)	.65	2.91 (1.61)	3.87 (1.42)	4.01 (1.32)	<.001

## Discussion

### Principal Findings

NLP of patient medication reviews identified 5 topics per ED drug. Sildenafil had 2 topics on treatment benefit (*sexual performance* and *erection sustainability*) and 1 topic on medication safety (*medication safety*). In contrast, tadalafil had 1 topic on treatment benefit (*sexual performance*) and 2 topics on medication safety (*mild medication safety* and *serious medication safety*).

*Erection sustainability* was additionally identified as a treatment benefit of sildenafil. Younger patients seemed to have received the most benefit from erection sustainability. The topic was more frequently mentioned (30/124, 24.2%) among younger patients than other age groups. Moreover, younger patients gave the highest satisfaction rating (4.37/5) when they mentioned *erection sustainability* as the primary topic compared with other primary topics. It has been known that sildenafil’s marketing strategy was to increase its consumer base by appealing to younger adults [30,31]. To that end, the drug seller must have succeeded in incepting the concept that sildenafil enhances sexual performance, something younger adults desire, rather than treating the medical problem of ED prevalent among older adults [33-35].

It is a bit surprising that the reviews on tadalafil did not reveal *erection sustainability* as a topic considering that the drug remains longer in the blood compared with sildenafil. Evidently, erection sustainability must have meant how long an erection can last during sexual intercourse rather than how long the drug remains in the blood. Perhaps, tadalafil users were more concerned about erection readiness rather than erection sustainability. For this reason, older adults who desire erection readiness more than erection sustainability were more satisfied with tadalafil than with sildenafil [36,37].

The topic identification of patient medication reviews successfully uncovered the marketing claims of each drug, that is, the *amber romance* topic had a list of words like “last,” “still,” and “weekend,” while the *blue magic* topic had a list of words like “hour,” “dose,” “minute,” and “rapid.” Eli Lilly, the tadalafil seller, knew that ED patients want sex to be more “natural” and therefore casted middle-aged actors in tadalafil commercials [38,39]. The commercials emphasized that the drug makes you ready whenever you feel like making love, which promotes romance over sexual acts. The seller even designed the pill to appear as a blown-up amber-colored heart. In contrast, Pfizer, the sildenafil seller, emphasized sexual performance over a romantic relationship. The seller incorporated a blue diamond shape into the pill design to make the drug look quite strong. These marketing claims are backed by some scientific evidence. The claim pertaining to *blue magic* is based on the pharmacokinetic property that the drug works rapidly and then clears out of the body with a half-life of 4 hours. On the other hand, the amber heart pill lasts for 3 days, which was promoted as a weekend pill where retaking the drug is not needed for successive sexual arousals for weekend romance.

The discovery that marketing claims are reflected in patient medication reviews suggests that ED drug users respond to marketing claims. The main goal of marketing is to identify who responds to commercial advertisements. In our study, the youngest age group was more satisfied with sildenafil than with tadalafil (score 3.73 vs 3.21). The youngest group was also more satisfied when *blue magic* was their primary topic rather than *amber romance*. These findings were reversed among the oldest group. Despite the greater uncertainty about the differentiation, both drugs seem to have successfully realized their respective marketing claims.

The numbers of topics related to medication safety were 2 for tadalafil and 1 for sildenafil. This indicates that safety concerning tadalafil has 2 subdimensions, one for *serious medication safety* and the other for *mild medication safety*, while sildenafil has 1 dimension called *medication safety*. Although tadalafil and sildenafil belong to the same class of PDE5 inhibitors, they clear out of the body differently; tadalafil lingers long in the blood, whereas sildenafil clears out of the body rapidly. Back pain and myalgia, which might be more prevalent among younger adults, result from PDE5 action [40]. Thus, it is likely that the lingering action of tadalafil could have aggravated the pain associated with PDE5 action [41].

Expectedly, patients who mentioned *serious medication safety* as the primary topic gave the lowest drug rating (2.44) compared with those who mentioned *medication safety* (3.30) or *mild medication safety* (3.52) as the primary topic. Among patients who had received ED drug therapy for less than 1 month, the primary topic of *serious medication safety* had the largest share (almost 40%). The share decreased to 10% among users who had used the drug for more than 1 year. It is worth noting that those who regarded *sexual performance* as the primary topic had a rating higher than 4.00 regardless of the time on tadalafil; however, among those with serious medication as the primary topic, the drug rating went up as the time on tadalafil increased. Logically, tadalafil users would stop taking the medication when they face a serious medication safety event. This explains why the proportion of patients who had used the ED drug for more than 1 year was lower for tadalafil than for sildenafil (187/919, 20.3% and 150/463, 32.4%, respectively).

Finally, the topic *alternative choices* was identified with regard to both drugs. It had a list of words like “Cialis,” “trial,” “another,” and “generic” for sildenafil and words like “Viagra,” “trial,” “Levitra,” and “generic” for tadalafil. It is certainly important for the patient to have access to alternative medications, especially since the high prices of ED drugs have been a burden on patients because of a lack of insurance coverage. In fact, patients frequently mentioned the generic versions that are 50 times cheaper than the branded pills [42]. Furthermore, the presence of the topic is aligned with the research in that one of the main reasons for risking to buy potentially counterfeit sexual stimulants, including Viagra and Cialis, is related to poor finance [43].

It is interesting why sildenafil users least frequently mentioned *alternative choices* as their primary topic, while tadalafil users mentioned it quite frequently (71/461, 15.4% vs 182/915, 19.9%). The alternative choices may not be as important to sildenafil users as they are to tadalafil users. Tadalafil users may have faced serious medication safety events (the largest share of primary topics: 244/915, 26.7%) and thus might have been motivated to talk about alternative choices. However, sildenafil users who less frequently (104/461, 22.6%) faced a medication safety event would have talked about it less frequently. Moreover, medication reviewers who had *alternative choices* as their primary topic were more satisfied with sildenafil than with tadalafil, except for the oldest group. The reviewers also gave better ratings to sildenafil than to tadalafil across multiple times on ED medication.

### Practice Implications

The identification of topics hidden in the patient reviews of ED drug therapy via topic modeling can have many applications. It can help evaluate whether the marketing claims have effectively targeted a specific group of people who desire a certain medication attribute for their health needs. It can also contribute to patient-centered care by informing health care providers of the different medication concerns facing individual patients taking ED drug therapy. Lastly, the study findings have documented the capabilities of topic modeling on SNS drug reviews in the areas of infodemiology/infoveilance of private and taboo topics. Topic modeling of ED drug reviews posted on SNSs can effectively reveal honest attitudes and opinions toward sexual needs not expressed in formal surveys. It could pave the way for topic modeling on SNS posts as an efficient social research tool to identify the needs of vulnerable populations whose opinions and orientations are not well accepted in society.

### Limitations

There may be biases that arise from using online reviews on social media. Online reviews may likely be posed by those who are eager to express their eccentricity. Therefore, it is likely they are not representative of the general public. In other words, the findings cannot be generalized to the public. However, the comparison between the 2 drugs may not have the limitation of selection bias since there appeared to be no systematic differences among the reviewers of the 2 ED drugs.

Patient medication experiences related to safety issues may have been exaggerated. It has been shown in previous research that a consumer’s motivation to review a product is to inform others to avoid a negative experience [44,45]. Moreover, despite filtering the reviews, unidentified spam reviews might have gone undetected. Unfiltered spam reviews can affect the study results by intentionally giving false positive or malicious negative opinions about the drugs [46,47].

Naming each topic identified was done subjectively based on each list of words in each topic. Therefore, topic names may not capture all the minute nuances contained in each list. Furthermore, the researchers’ subjectivity may have played an important role in extracting hidden topics since the number of topics is given by the authors. The optimal number of topics may vary based on specific criteria.

Despite the same data collection criteria, the number of patient medication reviews for sildenafil was almost half that for tadalafil. This may have resulted from the misaligned times between drug approval dates and SNS popularity [48]; drug reviews on SNSs were less popular when sildenafil was approved. In fact, ED was too sensitive to mention in public when sildenafil was first marketed. People became more comfortable with its discussion over time with continuous branding of ED as a medical problem to be treated [31,49]. Finally, tadalafil reviewers might have been more motivated to leave posts because they were more likely to mention medication safety than efficacy (ie, on medication safety, tadalafil had 2 topics while sildenafil had 1, and on efficacy, tadalafil had 1 topics while sildenafil had 2).

It is unlikely that the unbalanced number of patient medication reviews between the 2 drugs produced a bias in the study results. Because separate topic modeling was run for each drug set of reviews, the identification of topics would not be affected by the unbalanced number. However, it raises the question whether the number of sildenafil reviews was sufficient for topic modeling. It is reported that the sample size requirement for topic modeling varies with document characteristics, such as content heterogeneity and document length [50,51]. Patient medication reviews have a longer document length than typical tweets. They are also homogenous because they are from the patients who have taken medication for ED. It is reported that people with specific health problems provide informative and lengthy text data for health portals [52]. In addition, our study successfully identified 5 distinctive topics meeting the topic identification criteria [25]. Furthermore, a previous study successfully executed topic modeling based on less than 500 social reviews [53].

### Conclusion

The topics identified from patient medication reviews of ED drugs reflect drug labeling information, marketing claims, and comparative alternative choices facing patients in real-world practice. Topic modeling of natural language expressed in patient medication reviews can identify patient medication concerns, which are crucial for patient-centered prescription and medication counseling. Moreover, it supports that topic modeling on SNS posts is capable of uncovering hidden topics related to taboo or private behaviors.

## Abbreviations

ED: erectile dysfunction
FDA: Food and Drug Administration
LDA: latent Dirichlet allocation
NLP: natural language processing
PDE5: phosphodiesterase type 5
PRO: patient-reported outcome
SNS: social network service
